# Clinical validation of full HR-HPV genotyping HPV Selfy assay according to the international guidelines for HPV test requirements for cervical cancer screening on clinician-collected and self-collected samples

**DOI:** 10.1186/s12967-022-03383-x

**Published:** 2022-05-17

**Authors:** Alice Avian, Nicolò Clemente, Elisabetta Mauro, Erica Isidoro, Michela Di Napoli, Sandra Dudine, Anna Del Fabro, Stefano Morini, Tiziana Perin, Fabiola Giudici, Tamara Cammisuli, Nicola Foschi, Marco Mocenigo, Michele Montrone, Chiara Modena, Martina Polenghi, Luca Puzzi, Vjekoslav Tomaic, Giulio Valenti, Riccardo Sola, Shivani Zanolla, Enea Vogrig, Elisabetta Riva, Silvia Angeletti, Massimo Ciccozzi, Santina Castriciano, Maria Pachetti, Matteo Petti, Sandro Centonze, Daniela Gerin, Lawrence Banks, Bruna Marini, Vincenzo Canzonieri, Francesco Sopracordevole, Fabrizio Zanconati, Rudy Ippodrino

**Affiliations:** 1 Ulisse BioMed S.P.a, Area Science Park, SS 14, km 163.5, Trieste, Italy; 2 Molecular Genetics and Biotechnology PhD Study Programme, University of Nova Gorica, Nova Gorica, Slovenia; 3 Ginecologia Oncologica, IRCCS - Centro Di Riferimento Oncologico (CRO) (Istituto Nazionale Tumori – National Cancer Institute), Aviano, Italy; 4 Azienda Sanitaria Universitaria Giuliano Isontina UCO/SC Anatomia e Istologia Patologica, Cattinara Hospital, Trieste, Italy; 5 Department of Medicine, Surgery and Health Sciences, University of Trieste, Trieste, Italy; 6 Anatomia Patologica, IRCCS – CRO (Istituto Nazionale Tumori - National Cancer Institute), Aviano, Italy; 7 Institut Ruđer Bošković, Zagreb, Croatia; 8 Policlinico Universitario Campus Biomedico, Rome, Italy; 9 Copan Italia Spa, via F. Perotti 10, 25125 Brescia, Italy; 10 Institute of Maternal and Child Health, IRCCS Burlo Garofolo, Trieste, Italy; 11 Clinical Research Unit, Azienda Sanitaria Universitaria Giuliano Isontina, Trieste, Italy; 12 Cervical Cancer Screening Coordination Unit, Azienda Sanitaria Universitaria Giuliano Isontina, Trieste, Italy; 13 International Centre for Genetic Engineering and Biotechnology, Trieste, Italy

**Keywords:** Human Papillomavirus, HPV, Cervical cancer, HPV test, Clinical performance, Cervical cancer screening, Self-sampling, HPV genotyping, Meijer’s guidelines, VALHUDES

## Abstract

**Background:**

According to international guidelines, Human Papillomavirus (HPV) DNA tests represent a valid alternative to Pap Test for primary cervical cancer screening, provided that they guarantee balanced clinical sensitivity and specificity for cervical intraepithelial neoplasia grade 2 or more (CIN2+) lesions. The study aimed to assess whether HPV Selfy (Ulisse BioMed – Trieste, Italy), a full-genotyping HPV DNA test that detects and differentiates 14 high-risk HPV (HR-HPV) types, meets the criteria for primary cervical cancer screening described in the international guidelines, on clinician-collected as well as on self-collected samples.

**Methods:**

For each participant woman, consecutively referring to Azienda Sanitaria Universitaria Giuliano Isontina (Trieste, Italy) and CRO—National Cancer Institute (Aviano, Italy) for the cervical cancer screening program, the following samples were tested: (a) a clinician-collected cervical specimen, analyzed with the reference test (Hybrid Capture®2 test, HC2) and HPV Selfy; and (b) a self-collected vaginal sample, analyzed with HPV Selfy. Enrolled women were also asked to fulfill a questionnaire about self-sampling acceptability. As required by guidelines, a non-inferiority test was conducted to compare the clinical performance of the test under evaluation with its reference test.

**Results:**

HPV Selfy clinical sensitivity and specificity resulted non-inferior to those of HC2. By analysis of a total of 889 cervical liquid-based cytology samples from a screening population, of which 98 were from women with CIN2+, HPV Selfy showed relative sensitivity and specificity for CIN2+ of 0.98 and 1.00 respectively (non-inferiority score test: *P* = 0.01747 and *P* = 0.00414, respectively); the test reached adequate intra- and inter-laboratory reproducibility. Moreover, we demonstrated that the performance of HPV Selfy on self-collected vaginal samples was non-inferior to the performance obtained on clinician-collected cervical specimen (0.92 relative sensitivity and 0.97 relative specificity). Finally, through HPV Selfy genotyping, we were able to describe HPV types prevalence in the study population.

**Conclusions:**

HPV Selfy fulfills all the requirements of the international Meijer’s guidelines and has been clinically validated for primary cervical cancer screening purposes. Moreover, HPV Selfy has also been validated for self-sampling according to VALHUDES guidelines. Therefore, at date, HPV Selfy is the only full-genotyping test validated both for screening purposes and for self-sampling.

*Trial registration* ASUGI Trieste n. 16008/2018; CRO Aviano n.17149/2018

**Supplementary Information:**

The online version contains supplementary material available at 10.1186/s12967-022-03383-x.

## Background

Cervical cancer is the fourth most common tumor in women worldwide, with estimated 569.847 new cases and 311.365 related deaths per year [1]. In the last decades, cervical cancer-related mortality dramatically decreased, thanks to the widespread use of screening programs [2]. Cervical cytology has been used for years as standard test for cervical cancer screening [3]. However, it has some potential limitations: conventional staining procedure requires a considerable amount of time and consumables; smearing process of the Pap test is characterized by poor reproducibility [4]; errors in the interpretation of the results can be caused by blood and mucus, imperfection in the fixation or by a non-uniform distribution of cells on the slide [5]. Moreover, it requires a gynecologist (or midwife) to be performed and a cytologist to be analyzed, with an increase in costs and the necessity of a proper setting.

The etiological link between HR-HPV persistent infection and the development of high grade cervical dysplasia and cervical cancer is well known [6]. Therefore, HR-HPV DNA tests have been developed as an alternative strategy for cervical cancer screening, overcoming the potential limitations of cervical cytology. Four randomized controlled trials (SWEDESCREEN [7], POBASCAM [8], ARTISTIC [9] and NTCC [10]) demonstrated that the HPV-based screening helps to detect persistent high-grade cervical lesions before the conventional cytology, providing a 60–70% greater protection against invasive cervical carcinomas compared to Pap smear [11]. According to these findings, European countries are currently moving towards HR-HPV DNA test for cervical cancer screening, with a 5-years interval between each screening round for everyone with a cervix from age 25 until age 65. In Europe, most countries have implemented/are implementing an organized population-based program for cervical cancer screening, call/recall invitation system; however half countries still have in place opportunistic screening program depending on the initiative of the individual women and/or her doctor, and some countries still lack any screening program. In addition, according to the European Centre for Disease Prevention and Control, several countries had introduced HPV vaccination in females and males: indeed, nonavalent vaccine, licensed in 2015, helps protecting against 7 HR-HPV types (16, 18, 31, 33, 45, 52 and 58) and 2 low risk HPV types (6 and 11); however, HPV vaccine is not intended to replace screening tests, that remain an essential part of preventive health care.

HR-HPV DNA molecular tests are characterized by high sensitivity that allows to detect also clinically insignificant, transient infections, that will not lead to lesion development. Therefore, HR-HPV DNA tests with intended use for primary screening should guarantee a balanced clinical sensitivity and specificity in order to allow effective detection of cervical intraepithelial neoplasia grade 2 (CIN2) or more severe lesions (> CIN2) and minimize follow up procedures on HPV test-positive women without clinically meaningful disease (< CIN2). The most widely adopted and internationally accepted guidelines describing the minimum requirements of HR-HPV DNA tests for primary cervical cancer screening were introduced in 2009 (Meijer’s guidelines) [12]. Nevertheless, according to a 2020 review of commercially available assays by Poljak and colleagues, out of 254 HR-HPV DNA tests, only 13 have been clinically validated according to such guidance; 81.8% of HR-HPV DNA tests lack any published analytical and/or clinical evaluation and over 90% are not evaluated in line with consensus requirements that ensure safe use in clinical settings [13]. Similarly, the latest 2021 report of Gruppo Italiano Screening del Cervicocarcinoma (GISCI) reported 16 HPV assays validated for primary screening according to Meijer’s guidelines [14], whereas the 2020 report by European Society of Gynaecological Oncology (ESGO) indicated only 10 tests for screening purposes [15].

Moreover, the majority of commercially available HR-HPV DNA tests are compatible with clinician-collected cervical samples only [16, 17]. The opportunity of a self-collectable sample and effective test could help to recruit this “under-screened” population, especially in developing countries where lack of resources and psychological and cultural barriers can limit the access to gynecological services [18–24] Indeed, the effectiveness of cervical cancer screening programmes depends on women’s participation and coverage. Estimated screening coverage in European countries is 63%, ranging from 80% in Austria to less than 50% in Ireland; in developing countries instead the average screening coverage is only 19% [25]. However, to ensure safety of self-collection application, diagnostic tests should be specifically validated for this purpose and have this scope stated in their intended use. For proper clinical validation, the VALHUDES protocol offers guidance to compare the clinical sensitivity and specificity of a candidate HR-HPV assay on vaginal self-samples and first void-urine, collected in agreement with standardized protocols, with the same HR-HPV DNA tests on matched clinician-taken samples. However, at date, out of 254 very few tests have self-collection included in their intended use. Moreover, out of the 13 and 16 tests clinically validated for screening purposes according to Poljak’s review (2020) and GISCI report (2021) respectively, at date only two tests have been validated for primary screening with self-collected samples. In the list of ESGO (2020), out of 10 tests, none satisfies these criteria.

HPV Selfy (Ulisse BioMed, Italy) is a novel real time PCR-based assay, based on SAGITTA platform, a proprietary technology platform that allows to perform multiplex PCR through melting curve analysis using a single fluorescence channel. HPV Selfy is capable of simultaneous detection and genotyping of 14 HR-HPVs (HPV types 16, 18, 31, 33, 35, 39, 45, 51, 52, 56, 58, 59, 66 and 68) in a single reaction (full genotyping).

The aim of this study is to clinically validate HPV Selfy (Ulisse BioMed, Italy) on clinician-collected cervical specimen as well as on vaginal secretions. Therefore, if used for primary screening, HPV Selfy could not only offer compatibility with different collection methods but also provide additional epidemiological information about HPV types prevalence.

## Methods

### Study design

From September 2018 to September 2019, women consecutively referring to Azienda Sanitaria Universitaria Giuliano Isontina (ASUGI – Trieste, Italy) and to CRO—National Cancer Institute (Aviano, Italy) for the Italian region Friuli Venezia Giulia (FVG) cervical cancer screening programme were asked to participate to the study. A proper informed consent was signed by each enrolled woman. The demographic and clinical data of each patient enrolled were collected in a proper database.

At the time of the enrollment, the FVG cervical cancer screening programme was based on cytology every 3 years for women aged 25 to 64 years old. In case of any cytological abnormalities, a colposcopy (and eventually a colposcopy-guided biopsy) was recommended. In case of Atypical Squamous Cells of Undetermined Significance (ASC-US) on Pap smear, a triage HR-HPV DNA test was performed, and only women tested positive were sent to colposcopy. The FVG screening programme had a participation coverage of 66.6% (last data available 2016–2019; https://www.epicentro.iss.it/passi/dati/ScreeningCervicale). Pregnant women and women with current diagnosis of uterine, endometrial, vaginal, vulvar or ovarian cancers were not included in the present analysis. Those who used vaginal ovules, creams or had vaginal douching, sexual intercourses or menses in the three days prior the exam were excluded.

### Study procedures

After enrollment, women were asked to perform a self-sampling vaginal swab using a sterile dry flocked swab (FLOQSwabs®, Copan, Brescia, Italy) for a subsequent analysis with our real time PCR-based detection methodology, HPV Selfy. Self-collection was performed in a separate dressing room, immediately prior the gynecological visit. After the self-collection, a trained midwife or a gynecologist collected a cervical sample for the Pap smear using a cervical brush and rinsed the same cervical brush in 20 ml ThinPrep® PreservCyt media (Hologic, Marlborough, MA, USA) (ThinPrep) to assess HPV infection using the Hybrid Capture® 2 High- Risk HPV DNA test for comparison. After the sampling, self-collected dry swabs were stored at – 20 °C at the clinical sites and sent to Ulisse BioMed laboratories. Pap smears for standard cytological examination and ThinPrep vials for HC2 and HPV Selfy testing were sent to the local anatomic pathology department (UCO/SC Anatomia e Istologia Patologica, ASUGI Trieste, Italy). Histological analysis of colposcopy-guided biopsy was performed in case of cytological abnormalities. At the end of the procedure, women were asked to fill out an anonymous questionnaire on their opinions and preferences about cervical samplings.

### Hybrid capture 2, Pap test and histological analysis

Clinician-collected samples in ThinPrep media were analyzed using HC2 following manufacturer’s instructions [26]. HC2 is a CE IVD and FDA-approved test based on an in vitro nucleic acid hybridization assay with chemiluminescent signal amplification for detection of 13 High-Risk HPVs (HPV 16, 18, 31, 33, 35, 39, 45, 51, 52, 56, 58, 59 and 68) in cervical brush-collected samples [27]. The assay explois full genome RNA probes complementary to the HPV DNA, creating RNA/DNA hybrids. Then, the RNA/DNA hybrids are captured onto a solid phase coated with universal capture antibodies specific for RNA/DNA hybrids. The specimen matrix is then washed from the captured hybrids to remove inhibitors. During the signal amplification, captured RNA/DNA hybrids are detected with multiple antibodies conjugated to alkaline phosphatase. The signal resulting from the chemiluminescent reaction is read and the results are automatically interpreted.

HC2 was selected as comparator test, being the reference test according to Meijer’s guidelines [12].

Cervical smear slides were Pap-stained, and histo-technicians interpreted the results following the Bethesda 2001 classification [28].

### HPV Selfy

ThinPrep and self-collected swabs were tested using HPV Selfy. HPV Selfy is a novel in vitro diagnostic real time PCR-based test followed by melting curve analysis, utilizing a single fluorophore channel. The assay exploits an innovative technological platform for nucleic acid multiplex analysis detection. HPV Selfy is capable to detect and perform genotyping of 14 HR-HPV types (16, 18, 31, 33, 35, 39, 45, 51, 52, 56, 58, 59, 66 and 68) in a single real time PCR reaction. Melting curve analysis step generates distinctive melting peaks for each single HPV genotype present in the sample, through a proprietary reagent and process named as Sagitta melting fingerprinting technology. This is possible through the amplification of HPV types-highly specific regions spanning the entire viral genomes, which generate amplicons with a unique melting temperature. Viral DNA Extraction was performed with ReliaPrep™ Blood gDNA Miniprep System (Promega; Madison, Wisconsin, United States); alternatively, samples were pre-treated with Ulisse Faster DNA (Ulisse BioMed, Italy), a reagent that allows the lab user to skip DNA extraction and to directly load in the PCR reaction the raw samples after a brief pretreatment, thus saving time and costs. HPV Selfy test includes a human DNA amplification control (Haemoglobin subunit beta) to evaluate sample quality, thereby reducing the risk of false-negative results. Limit of Detection was defined by testing serial dilutions plasmids containing HPV full reference genomes at known concentration (kindly provided by Prof. Carina Eklund, International Human Papillomavirus Reference Center, Karolinska Institute, Sweden); analytically, HPV Selfy is able to detect down to 100 genome copy number per reaction in average, as declared by manufacturer’s instructions for use. The threshold used for the clinical study was adjusted to detect clinically relevant infections and to achieve adequate clinical specificity and sensitivity [29].

Analysis was performed according to manufacturer's protocol, using a QuantStudio 5 Real Time PCR machine (Thermo Fischer Scientific; Waltham, Massachusetts, United States). For inter-laboratory reproducibility, the samples were tested at the local Anatomic Pathology Department (UCO/SC Anatomia e Istologia Patologica, ASUGI Trieste, Italy) and Medichrom (Pistoia, Italy) as well.

### HPV genotyping

A subpopulation of samples was analyzed with a CE-IVD test able to genotype high and low risk HPVs: CLART® HPV 2 (Genomica, Madrid, Spain) (CLART). CLART detects 14 HR-HPV types (16, 18, 31, 33, 35, 39, 45, 51, 52, 56, 58, 59, 66, and 68) and 21 low-risk and probable high-risk HPV types (6, 11, 40, 42, 43, 44, 54, 61, 62, 70, 71, 72, 81, 83, 84, 85, 89, 26, 53, 73 and 82) using a PCR amplification followed by a microarray hybridization assay [30].

### Statistical analysis

HPV Selfy results were considered positive when at least one of the 14 HR-HPV types was detected with a fluorescence threshold of 710.000; HC2 results were considered positive when the Relative Light Units/cutoff (RLU/CO) values were 1 [26], and CLART results were considered positive only for the detection of the 14 HR-HPV types included in HPV Selfy HPV test (i.e., samples detected by CLART as low-risk or probable high-risk HPV were considered as negative).

As indicated by Meijer’s guidelines, to compare the clinical sensitivity and specificity for ≥ CIN2 of HPV Selfy to that of HC2, a non-inferiority score test with a power of at least 80% was performed. The relative sensitivity and specificity thresholds used were 0.90 and 0.98 respectively, as proposed in the published guidelines [12, 31]; a *P* value (*P*) < 0.05 was considered statistically significant.

The Cohen’s kappa statistic was used to test intra- and inter-laboratory reproducibility. The candidate test for Meijer’s guidelines should show a percentage of agreement with a lower confidence bound not less than 87% (in a population of 500 samples including 30% positives).

As indicated by VALHUDES protocol, to compare the clinical sensitivity and specificity of HPV Selfy for self-collected samples vs clinician-collected samples, alpha = 0.05, beta = 0.20, lower confidence interval for relative sensitivity and specificity (index/comparator) = 0.90 and 0.95, respectively, was computed.

Agreement was assessed using Cohen's Kappa statistic (κ). Interpretation of the κ values followed the proposed standards of Landis and Koch: slight (0–0.20); fair (0.21–0.40); moderate (0.41–0.60); substantial (0.61–0.80); and almost perfect (0.81–1.00).

Statistical analysis was conducted with R version 3.5.0 and https://www.graphpad.com/quickcalcs/kappa2/;s*P* < 0.05 were considered as statistically significant.

## Results

During the study period, a total of 1234 women agreed to be enrolled in the present study and properly signed the informed consent. Among them, 29 women were excluded for incomplete samples collection, samples non properly stored before analysis, or lack of Pap smear result.

### Clinical validation of HPV Selfy on clinician-collected ThinPrep samples (Meijer’s guidelines)

We aimed to validate HPV Selfy for clinician-collected cervical specimen according to Meijer’s guidelines. 98 women ≥ 30 years old had a biopsy-diagnosed high grade cervical lesion (CIN2 or worse, defined later as “CIN2 + Group”) at the time of the sample collection or within 2 years of follow-up; whereas 791 women ≥ 30 years old had two consecutive normal cytology results and without evidence of CIN2+ within 2 years of follow-up (defined later as “Control Group”).

Therefore, a total of 889 women were included in the study population for evaluation of performance according to Meijer’s guidelines; the main clinical and demographic characteristics of this cohort are reported in Table 1.

**Table 1:** Description of demographic characteristics of the study cohort. Main characteristics of the study population taken into account for validation according to Meijer’s guidelines were recorded (age; smoke; menopause; contraceptive; concurrent genital infections)

	Number | (889)	%
Age		
30–39 years	190	21.4
40–49 years	303	34.1
50–59 years	299	33.6
Over 60 years	97	10.9
Smokers	249	28.0
Menopause	320	36.0
Contraceptive	201	22.6
Concurrent lower genital tract infections (e.g.: condidiasis, BV, chlamydia, herpes genitalis)	42	4.7

Results of clinical validation are reported in Table 2. HPV Selfy was positive for 79 women with CIN2+, resulting in an absolute clinical sensitivity for CIN2+ of 0.81 (79/98; 95% confidence interval [CI] 0.71 to 0.88), in comparison with 0.83 absolute sensitivity of HC2 (81/98; 95% CI 0.74 to 0.89). Relative sensitivity of HPV Selfy in comparison with HC2 was 0.98.

**Table 2:** Clinical validation of HPV Selfy for primary HPV screening according to Meijer’s guidelines. HPV Selfy assay vs HC2 results performed on 889 ThinPrep samples from population-based screening stratified by case–control status

Controls		HC2		Total
		Negative	Positive	
**HPV Selfy**	Negative	723	22	745
	Positive	19	27	46
	Total	742	49	791

CIN2+				
**HPV Selfy**	Negative	16	3	19
	Positive	1	78	79
	Total	17	81	98

The absolute clinical specificity for CIN2+ was 0.94 for HPV Selfy (745/791; 95% CI, 0.92 to 0.96), in comparison with 0.94 of HC2 (742/791; 95% CI 0.92 to 0.95). Relative specificity of HPV Selfy in comparison with HC2 was 1.00.

Relative clinical sensitivity and specificity values of HPV Selfy were compared to those of HC2 using a non-inferiority score test [12, 31] with a relative sensitivity threshold for CIN2+ of 0.90 and a relative specificity threshold for CIN2 + of 0.98 using HC2 as a reference—as indicated in Meijer’s guidelines. The non-inferiority score test demonstrated that HPV Selfy clinical sensitivity and specificity for CIN2+ were non-inferior to those of HC2 (T = 2.109; *P* = 0.01747 for sensitivity, T = 2.640 *P* = 0.00414 for specificity).

Intralaboratory reproducibility was determined by analyzing 521 randomly selected samples, of which 157 were HC2 positive (30.13%), twice, in the same lab. Overall concordance observed was 94.6% with a kappa value of 0.87 (95% CI 0.83 to 0.92) (Additional file 1: Table S1). Interlaboratory reproducibility was determined by analyzing 500 randomly selected samples, of which 150 HC2 positive (30%), analyzed by two independent laboratories. Concordance observed was 93.6% with a kappa value of 0.85 (95% CI 0.80 to 0.90) (Additional file 1: Table S2). Cohen’s kappa values associated to intra- and inter-laboratory reproducibility experiments were greater than the minimum required value for a clinical test [12] and proved that the test under evaluation produces reliable results. In conclusion, HPV Selfy fully satisfies Meijer’s requirements for primary HPV screening.

### Comparison between DNA extraction and Ulisse Faster treatment

HPV Selfy was performed also directly, without DNA extraction, in combination with Ulisse Faster DNA reagent, on a subpopulation of 144 clinician-collected cervical samples, of which 72 HPV Selfy-negative and 72 HPV Selfy-positive randomly selected samples. The agreement between the assays performed on samples after DNA extraction or after Ulisse Faster pretreatment was perfect (100%; K of Cohen: 1) (Table 3).

**Table 3:** Comparison of HPV Selfy results executed upon DNA extraction or Ulisse Faster treatment. Comparison between HPV Selfy was performed on samples after DNA extraction or after Ulisse Faster pretreatment, in a population of 144 samples

		Extracted DNA		Total
		Negative	Positive	
**Ulisse Faster treated samples**	Negative	72	0	72
	Positive	0	72	72
	Total	72	72	144

### Clinical validation of HPV Selfy on self-collected vaginal samples (VALHUDES)

Next, we aimed at evaluating HPV Selfy performance on self-collected samples, as indicated by VALHUDES protocol. Hence, we needed to assess whether HPV testing on vaginal self-samples was as accurate as HPV testing on a cervical sample taken by a clinician. To do so, we identified 119 CIN2+ cases (age 25–65 years) and 791 ≤ CIN1 cases, for which we had available paired cervical specimen and self-collected vaginal samples.

HPV Selfy testing in self-collected samples was found similarly sensitive (88/96; relative sensitivity 0.92; 95% CI 0.84–0.96) and specific (724/745; relative specificity 0.97; 95% CI 0.96–0.98) to detect CIN2+ in the total study population (Table 4), in comparison with HPV Selfy performed on paired ThinPrep.

**Table 4:** Clinical validation of HPV Selfy for self-collection according to VALHUDES indications. Results of HPV Selfy assay performed on self-collected vaginal specimen vs ThinPrep cervical samples, performed on 910 paired samples from population-based screening stratified by case–control status

Controls		HPV Selfy (clinician-collected cervical samples)		Total
		Negative	Positive	
**HPV Selfy (self-collected vaginal samples)**	Negative	708	16	724
	Positive	37	30	67
	Total	745	46	791

CIN2+				
**HPV Selfy (self-collected vaginal samples)**	Negative	18	13	31
	Positive	5	83	88
	Total	23	96	119

Thus, HPV Selfy assay fulfills VALHUDES requirements for use of HR-HPV DNA tests on self-collected samples according to non-inferiority analysis (relative sensitivity > 0.90 with T = 3.62, *p* = 0.00015; relative specificity > 0.95 with T = 6.60, *p* < 0.00001).

Secondary objectives of VALHUDES protocol include the assessment of the absolute accuracy of HR-HPV DNA test applied according to the sampling device and the proportion of adequate samples as determined by amplification of an internal control (a ubiquitous human gene). HPV Selfy assay provides a human beta-globin internal control, used to evaluate sample quality. In the whole study cohort, mean Ct value for the human beta-globin internal control for the HPV Selfy test on self-collected samples, obtained with the direct analysis on self-collected samples, was 26.1 Ct (median value 25.9 Ct, maximum 30.7 Ct, minimum 16.5 Ct). In the subgroup of women with biopsy-diagnosis of cervical lesions CIN2+, the same analysis resulted in 26.7 Ct (median value 26.5 Ct, maximum 30.7 Ct, minimum 24.2 Ct). This means that all women were able to self-collect a similar amount of specimen, confirming self-collected samples’ quality adequacy and so the easiness of the self- sampling procedure using the FLOQSwabs® (Copan, Brescia, Italy).

### Analysis of discordant samples with third CE-IVD test

The present clinical study highlighted the presence of several discordant samples. Thus, we selected a third CE-IVD test, CLART® HPV 2, to test a subpopulation of 40 randomly selected self-collected samples that showed discordant results in comparison with HC2 reference test (regardless of age and cytology status). Interestingly, the CLART test results agreed with those of the HPV Selfy test performed on the same self-collected samples with an overall concordance of 92.5% (Cohen’s Kappa index: 0.85, almost perfect agreement), indicating that, in the large majority of cases, HPV Selfy results were correct and HC2 results were false (Table 5).

**Table 5:** Comparison between HPV Selfy and CLART result on a subpopulation of discordant samples (self-collected samples). A subpopulation of HPV selfy/HC2 discordant samples was tested with CLART test

Discordant samples	CLART		Total
	Negative	Positive	
**HPV Selfy**			
Negative	20	0	20
Positive	3^#^	17	20
Total	23	17	40

^#^ One of these 3 samples was positive to a low-risk HPV (HPV81) according to CLART test

Moreover, in the cases where we obtained discordant results with HPV Selfy in both specimen (cervical specimen and self-collected samples) in comparison to HC2, CLART test agreed with HPV Selfy in 100% of cases (21/21; Cohen’s Kappa index: 1, perfect agreement) (Table 6).

**Table 6:** Discordant samples analysis with a third HPV assay (CLART). Comparison of the four tests results on the different biological specimen tested with the different assays

*n*	Clinician-collected cervical samples		Self-collected vaginal samples	
	HPV Selfy	HC2	HPV Selfy	CLART
4	Positive	Negative	Positive	Positive
17	Negative	Positive	Negative	Negative
2	Negative	Negative	Positive	Negative
1	Positive	Positive	Positive	Negative
3	Positive	Positive	Negative	Negative
13	Negative	Negative	Positive	Positive

Additionally, we found two groups of samples with concordant opposite results in the two distinct samples: 13 women were negative with both test (HPV Selfy and HC2) at the cervix and positive with both test at the vaginal specimen (CLART and HPV Selfy), whereas 3 women were positive with both test (HPV Selfy and HC2) at the cervix and negative with both test at the vaginal specimen (CLART and HPV Selfy) (Table 6). This reflects the fact that in certain cases HPV could differentially infect different sites of the vaginal tract in which the sample is collected [32].

### Genotyping analysis

In order to evaluate HPV Selfy genotyping capability, 59 randomly selected HPV Selfy positive samples (regardless of age and cytology status) were tested also with CLART test, able to provide genotyping information. CLART identified 88 different viral infections, whereas 81 infections were found by HPV Selfy. Overall, the HPV genotyping agreement between the two tests was very good. In this subpopulation, CLART test detected fewer HPV68 and HPV39 infections than the HPV Selfy HPV test, while HPV Selfy detected fewer HPV59 and HPV66 infections (Table 7). We repeated HPV Selfy on the same samples in order to estimate genotyping reproducibility, and we obtained substantial to perfect agreement with type-specific kappa in a range from 0.73 to 1.00 (Additional file 1: Table S3). A general limitation of HPV genotyping studies is often represented by HPV-positive samples size, since some strains could not be enough represented in the analyzed population. Regarding HPV Selfy, additional data deriving from other studies, not yet published, are in the process of being analyzed in order to enhance statistical significance of HPV genotyping.

**Table 7:** Genotyping analysis of 59 HPV Selfy positive women tested with CLART HPV Test. A total of 81 HR-HPV types were detected with HPV Selfy and 88 HR-HPV types were detected with CLART. The table show high agreement in test genotyping

HR-HPV Types	HPV Selfy		CLART	
	n infections detected	%	n infections detected	%
16	19	23.5	19	23.5
18	0	0.0	0	0.0
31	14	17.3	14	17.3
33	3	3.7	3	3.7
35	3	3.7	3	3.7
39	5	6.2	5	6.2
45	0	0.0	0	0.0
51	4	4.9	4	4.9
52	7	8.6	7	8.6
56	4	4.9	4	4.9
58	7	8.6	7	8.6
59	0	0.0	0	0.0
66	10	12.3	10	12.3
68	5	6.2	5	6.2
Total	81	100.0	81	100.0

**Fig. 1:**
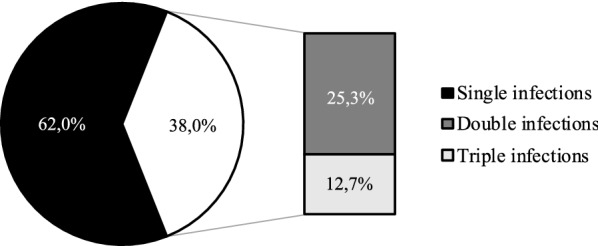
Analysis of HPV infections in CIN2+ Group. HPV infections were subdivided in single type infections and coinfections (n = 79 HPV-positive women)

**Fig. 2:**
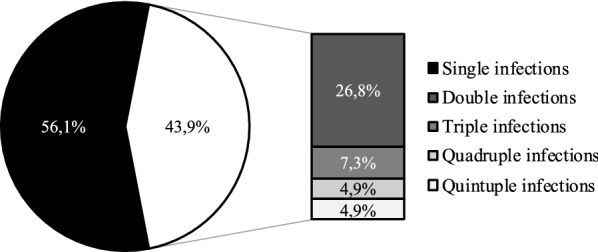
Analysis of HPV infections in Control Group. HPV infections were subdivided in single type infections and coinfections (n = 41 HPV-positive women)

Once confirmed the reliability of HPV Selfy genotyping, we proceeded to analyze cervical HPV infections associated with histologically confirmed CIN2+ lesions and cervical HPV infections not associated with high grade lesions, detected in the Control Group. In the CIN2+ Group, where 79 women were positive to HPV Selfy, we detected 49 single HPV type infections (62% of infected women) and 30 coinfections (38%), of which 20 double infections (25.3%) and 10 triple infections (12.7%) (Fig. 1). In the Control group, where 41 women resulted positive to HPV Selfy, we detected 23 single type infection (56.1%) and 18 coinfections (43.9%), of which 11 double infections (26.8%), 3 triple infections (7.3%), 2 quadruple infections (4.9%) and 2 quintuple infections (4.9%) (Fig. 2).

**Fig. 3:**
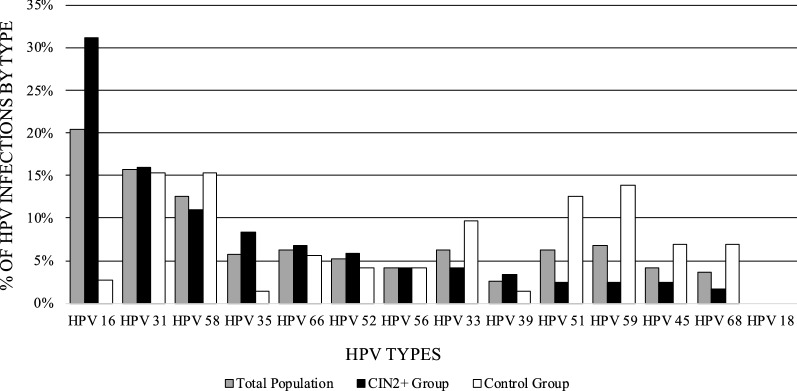
HPV prevalence in infections associated or not with CIN2+ lesions. We calculated HPV types frequency considering genotyping data obtained through HPV Selfy test. HPV types frequency observed in women without a histological diagnosis of high-grade cervical lesions (Control Group) is plotted with white bars (n = 72 infections); HPV types frequency observed in women positive for high-grade cervical lesions (CIN2+ Group) is plotted with black bars (n = 119 infections), versus HPV distribution in the total population (plotted with dark grey bars; n = 191 infections)

HPV Selfy genotyping identified that the most frequent HPV type present in the CIN2+ population was HPV16, followed by HPV31 and interestingly, HPV58, whereas in the < CIN2 population the most frequent were HPV31, HPV58 and HPV59 even though the prevalence strains were more homogeneously distributed (Fig. 3). Interestingly, no HPV18 (one of the most frequent HPV type worldwide reported in scientific literature) infections were detected by HPV Selfy in both populations, and only one was detected by CLART.

### Questionnaire results

**Fig. 4:**
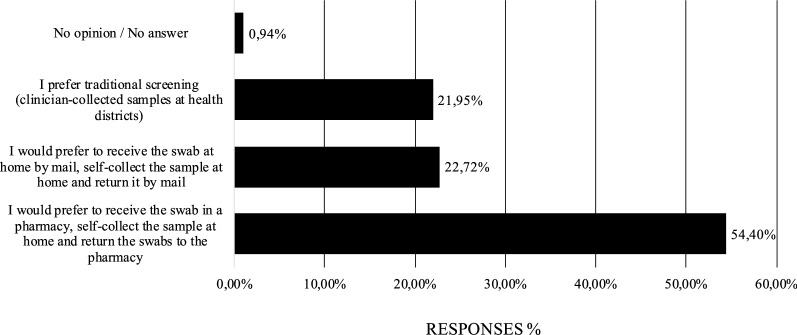
Response percentage regarding possible interests in future cervical cancer screening based on self-collection according to different modalities. According to the survey, a preference for picking-up and returning the swab at the pharmacies was recorded, compared to home-mailing of the swab (n = 1032 completed questionnaires)

All the women enrolled were asked to fulfill an anonymous questionnaire about self-sampling acceptability; we received 1032 completed questionnaires that were analyzed. We confirmed high acceptability to women for an HPV self- sampling screening compared with the Pap test control arm, as shown in other studies [33, 34]. Results of the survey are summarized in Table 8. At the question “Was the self-sampling easy to do?”: 98.26% answered yes. Additionally, 74.61% found self-collection less invasive, less painful and less embarrassing; 68.99% would prefer the self-collection method for the next screening program. On the contrary, 19.09% preferred clinician-sampling at health district clinics, mostly because they were concerned about self-sampling reliability and preferred to rely on midwives, while 9.69% had no preferences for sampling procedures in the future tests. Finally, 90.31% women said that they would welcome the possibility to perform self-sampling also at home (Table 8). 54.40% would prefer to receive the swab and return the self-collected sample to the pharmacies, as already happens for colorectal screening in Italy [35] (Fig. 4). No significant differences in the correlation between the age group and the ease of performing the self-sampling procedure were observed (*P* = 0.12), whereas we observed a greater preference for the self-sampling-based screening in younger women (25–35 years group, *P* = 0.02) (data not shown).

**Table 8:** Main results from the survey. According to the survey, high acceptability of the self-sampling procedure was recorded (n = 1032 completed questionnaires)

Question	Answer			
	Yes	No	No opinion	No answer
Was the self-sampling easy to do?	98.26%	1.55%	–	0.19%
Was the self-sampling less annoying if compared to the one performed by clinicians?	74.61%	8.72%	15.41%	1.26%
Would you prefer the self-collection method for the next screening program?	68.99%	19.09%	9.69%	2.23%
Would you perform self-sampling at home?	90.31%	8.43%	–	1.26%

## Discussion

This is the first clinical study describing clinical performance of HPV Selfy, a real time PCR assay able to detect 14 HR-HPV while performing full HR-HPV genotyping in a single reaction.

In this study, we compared the clinical performance of HPV Selfy on ThinPrep with that of HC2 in a cohort of screening participants. Since the assay meets the criteria for clinical equivalence and reproducibility of the international guidelines, HPV Selfy can be considered clinically validated for cervical screening purposes.

In the same study, HPV Selfy has been also validated on self-collected vaginal samples for screening purposes, satisfying the requirements indicated by VALHUDES protocol [36]. HPV self-sampling can be performed by using a simple vaginal swab, an easy and non-invasive self-sampling device [37]. Therefore, HPV self-sampling could be an attractive solution to increase women’s participation in opportunistic cervical cancer screening, regardless of age, education level, and other possible social parameters [38, 39]. HPV Selfy is one of the three assays worldwide that can claim the self-collection for primary screening purposes in its intended use. This can be of particular interest in the post-COVID19 era, where self-sampling represents a timely, accessible, safe and cost-effective strategy to efficaciously screen women while keeping social distancing [54].

Moreover, we demonstrated that HPV Selfy can be executed without DNA purification, in combination with Ulisse Faster DNA reagent, ensuring a significant reduction of analysis time and cost. HPV Selfy test has been designed to being suitable also for low-income settings, where cervical cancer is still a diffused plague given the lack of sustainable screening tests.

In conclusion, HPV Selfy presents several advantages compared to other validated assays, since it directly provides genotyping data of 14 HR-HPV types. At date, HPV genotyping is not suggested by most primary cervical screening guidelines, even though in certain countries HPV16 and HPV18 genotyping is used for differential triage. However, it is well-known that full HR-HPV genotyping is useful to verify the persistence of a specific HR-HPV type, that is associated with higher risk to develop a CIN2+ lesions. In addition, HR-HPV genotyping information is needed to assess the epidemiological distribution of HR-HPV types over time in a certain area, also taking into account the impact of local HPV vaccination campaigns, in order to produce scientific and epidemiological knowledge useful to define new cervical cancer screening policies.

## Conclusions

In conclusion, this study demonstrates that the full HR-HPV genotyping test HPV Selfy is clinically non-inferior to HC2. The clinical performance and reproducibility of the assay meet the international criteria for HR-HPV DNA test validation for cervical cancer screening purposes according to Meijers’s guidelines [12]. Moreover, we demonstrated that HPV Selfy has been also validated on self-collected samples for primary screening purposes, satisfying the requirements indicated by VALHUDES protocol [36].

## Supplementary Information

**Additional file 1: Table S1.** Intralaboratory reproducibility of HPV Selfy according to Meijer’s guidelines. HPV Selfy assay was performed twice on a subpopulation of 521 samples of which 157 samples positive to HC2. Overall concordance observed was 94.6% (kappa value of 0.87). **Table S2.** Interlaboratory reproducibility of HPV Selfy according to Meijer’s guidelines. HPV Selfy assay was performed in another laboratory (MediChrom) on a subpopulation of 500 samples of which 150 samples positive to HC2. Overall concordance observed was 93.6% (kappa value of **0.85). Table S3.** Intra-laboratory reproducibility of genotype findings of HPV Selfy. Data are presented as number of each genotype detected in each run (i.e. run1 and/or run 2), and numbers do not count up to the total number of HR-HPV positive samples due to multiple infections.

## Data Availability

All data generated or analysed during this study are included in this published article [and its additional files].

## Abbreviations

HPV: Human papillomavirus
PCR: Polymerase chain reaction
ASUGI: Azienda Sanitaria Universitaria Giuliano Isontina
HR-HPV: High-risk human papillomavirus
OR: Odd ratios
CIN: Cervical intraepithelial neoplasia
CE-IVD: CE marked-in vitro diagnostics
CI: Confidence interval
Ct: Cycle threshold
