# Correction: Clinical validation of full HR-HPV genotyping HPV Selfy assay according to the international guidelines for HPV test requirements for cervical cancer screening on clinician-collected and self-collected samples

**DOI:** 10.1186/s12967-023-03882-5

**Published:** 2023-01-26

**Authors:** Alice Avian, Nicolò Clemente, Elisabetta Mauro, Erica Isidoro, Michela Di Napoli, Sandra Dudine, Anna Del Fabro, Stefano Morini, Tiziana Perin, Fabiola Giudici, Tamara Cammisuli, Nicola Foschi, Marco Mocenigo, Michele Montrone, Chiara Modena, Martina Polenghi, Luca Puzzi, Vjekoslav Tomaic, Giulio Valenti, Riccardo Sola, Shivani Zanolla, Enea Vogrig, Elisabetta Riva, Silvia Angeletti, Massimo Ciccozzi, Santina Castriciano, Maria Pachetti, Matteo Petti, Sandro Centonze, Daniela Gerin, Lawrence Banks, Bruna Marini, Vincenzo Canzonieri, Francesco Sopracordevole, Fabrizio Zanconati, Rudy Ippodrino

**Affiliations:** 1 Ulisse BioMed S.p.A, Area Science Park, SS 14, km 163.5, Trieste, Italy; 2 Molecular Genetics and Biotechnology PhD Study Programme, University of Nova Gorica, Nova Gorica, Slovenia; 3 Ginecologia Oncologica, IRCCS—Centro Di Riferimento Oncologico (CRO) (Istituto Nazionale Tumori—National Cancer Institute), Aviano, Italy; 4 Azienda Sanitaria Universitaria Giuliano Isontina UCO/ SC Anatomia e Istologia Patologica, Cattinara Hospital, Trieste, Italy; 5 Department of Medicine, Surgery and Health Sciences, University of Trieste, Trieste, Italy; 6 Anatomia Patologica, IRCCS—CRO (Istituto Nazionale Tumori - National Cancer Institute), Aviano, Italy; 7 Institut Ruđer Bošković, Zagreb, Croatia; 8 Policlinico Universitario Campus Biomedico, Rome, Italy; 9 Copan Italia S.p.A., via F. Perotti 10, 25125 Brescia, Italy; 10 Institute of Maternal and Child Health, IRCCS Burlo Garofolo, Trieste, Italy; 11 Clinical Research Unit, Azienda Sanitaria Universitaria Giuliano Isontina, Trieste, Italy; 12 Cervical Cancer Screening Coordination Unit, Azienda Sanitaria Universitaria Giuliano Isontina, Trieste, Italy; 13 International Centre for Genetic Engineering and Biotechnology, Trieste, Italy

**Correction: Journal of Translational Medicine (2022) 20:231** 10.1186/s12967-022-03383-x

Following publication of the original article [1], we have been notified that the below-mentioned Correction article text should be published.

“We recently carried out a clinical validation of full high-risk HPV genotyping HPV Selfy assay [1] according to the international guidelines for DNA HPV test requirements for cervical cancer screening [2] as well as according to the reference protocol for validation of HPV assays for cervical cancer screening on self‑collected samples, i.e. the VALHUDES protocol [3], and we concluded that: (i) HPV Selfy is a high-risk HPV DNA test clinically validated for cervical cancer screening according to Meijer’s guidelines; and (ii) the performance of HPV Selfy on self-collected vaginal samples was non-inferior to the performance obtained on clinician-collected cervical specimens, implying that HPV Selfy is a test clinically validated for cervical cancer screening on self-collected samples.

Our paper demonstrated the non-inferiority of the performance of HPV Selfy obtained on self-collected vaginal samples applying the non-inferiority test recommended by the VALHUDES protocol [4] and, unfortunately, a statistical error has been made.

We noticed that we inverted two numbers applying the recommended formula to assess the non-inferiority of the sensitivity and specificity recorded on self-collected vaginal samples leading to incorrect results, as noticed by Marc Arbyn et al. in their letter to the Editor [5]. In particular, the number of subjects with discordant “self+ /clinician−” and “self−/clinician+” results (b and c cells in Table 4 of the paper) in the recommended formula for comparison of matched proportions were switched. Marc Arbyn et al. have claimed that the error, if corrected, would yield p values > 0.05, implying the inferiority of the specificity of HPV Selfy assay obtained on self-collected vaginal samples. Marc Arbyn et al. in their letter to the Editor stated that correct data entry would have generated non-inferiority p values 0.35 and 0.81 for sensitivity and specificity, respectively, and included in their letter to the Editor the detailed computation of the relative specificity. However, the recalculation of the relative specificity made by Marc Arbyn et al. was flawed by a statistical error: they used a δ_0_ equal to 0.98 instead of 0.95 yielding reported T equal to − 0.86 that implies p value > 0.05. The correct calculation using δ_0_ equal to 0.95 would quantify T equal to 2.11 implying p value < 0.05. This leads to the conclusion that still, relative specificity on self-samples is indeed significantly non-inferior to that observed in paired clinician-collected samples.

**Table 4:** Clinical validation of HPV Selfy for self-collection according to VALHUDES indications

Controls	HPV Selfy (clinical collected cervical samples)		Total
	Negative	Positive	
HPV Selfy (self collected vaginal samples)			
Negative	707	15	722
Positive	38	31	69
Total	745	46	791
CIN2+			
HPV Selfy (self collected vaginal samples)			
Negative	18	8	26
Positive	5	88	93
Total	23	96	119

Results of HPV Selfy assay performed on self-collected vaginal specimen vs ThinPrep cervical samples, performed on 910 paired samples from population-based screening stratified by case-control status

By reviewing the aforementioned calculations, we also noticed that a wrong test cut-off for sample positivity assessment has been applied while interpretating the results obtained on the self-collected vaginal samples. By applying the correct positivity cut-off for each sample type, we obtained the correct Table 4, the correct Results section of the Abstract and the correct paragraph titled “Clinical validation of HPV Selfy on self‑collected vaginal samples (VALHUDES)” that appear below with text amendments highlighted in bold italic. Additionally, there was a typo in Table 7 (a column was duplicated twice), this has been corrected; the correct Table 7 appears below.

**Table 7:** Genotyping analysis of 59 HPV Selfy positive women tested with CLART HPV test

HR-HPV types	HPV Selfy		CLART	
	n infections detected	%	n infections detected	%
16	21	23.9	19	23.5
18	1	1.1	0	0.0
31	15	17.0	14	17.3
33	4	4.5	3	3.7
35	4	4.5	3	3.7
39	3	3.4	5	6.2
45	0	0.0	0	0.0
51	5	5.7	4	4.9
52	6	6.8	7	8.6
56	3	3.4	4	4.9
58	7	8.0	7	8.6
59	5	5.7	0	0.0
66	14	15.9	10	12.3
68	0	0.0	5	6.2
Total	88	100.0	81	100.0

A total of 81 HR-HPV types were detected with HPV Selfy and 88 HR-HPV types were detectd with CLART. The table show high agreement in test genotyping

In conclusion, we regret any inadvertent errors in our paper and we state that the corrections made allow to confirm the scientific conclusions of the article and, so, that the performance of HPV Selfy on self-collected vaginal samples was non-inferior to the performance obtained on clinician-collected cervical specimens and, consequently, that HPV Selfy is a test clinically validated for cervical cancer screening on self-collected samples. The original article has been updated.

**Abstract section:**

**Results**

HPV Selfy clinical sensitivity and specificity resulted non-inferior to those of HC2. By analysis of a total of 889 cervical liquid-based cytology samples from a screening population, of which 98 were from women with CIN2+, HPV Selfy showed relative sensitivity and specificity for CIN2+ of 0.98 and 1.00 respectively (non-inferiority score test: P = 0.01747 and P = 0.00414, respectively); the test reached adequate intra- and inter-laboratory reproducibility.

Moreover, we demonstrated that the performance of HPV Selfy on self-collected vaginal samples was non-inferior to the performance obtained on clinician-collected cervical specimen (***0.97*** relative sensitivity and 0.97 relative specificity). Finally, through HPV Selfy genotyping, we were able to describe HPV types prevalence in the study population.

**Clinical validation of HPV Selfy on self‑collected vaginal samples (VALHUDES)**

Next, we aimed at evaluating HPV Selfy performance on self-collected samples, as indicated by VALHUDES protocol. Hence, we needed to assess whether HPV testing on vaginal self-samples was as accurate as HPV testing on a cervical sample taken by a clinician. To do so, we identified 119 CIN2+ cases (age 25–65 years) and 791 ≤ CIN1 cases, for which we had available paired cervical specimen and self-collected vaginal samples.

HPV Selfy testing in self-collected samples was found similarly sensitive (***93***/***96***; relative sensitivity ***0.97***; 95% CI ***0.90***–***1.04***) and specific (***722***/745; relative specificity 0.97; 95% CI ***0.95***–***0.99***) to detect CIN2+ in the total study population (Table 4), in comparison with HPV Selfy performed on paired ThinPrep. Thus, HPV Selfy assay fulfills VALHUDES requirements for use of HR-HPV DNA tests on self-collected samples according to non-inferiority analysis (relative sensitivity > 0.90 with T = ***1.70***, p = ***0.044***; relative specificity > 0.95 with T = ***1.87***, p =***0.031***).

Secondary objectives of VALHUDES protocol include the assessment of the absolute accuracy of HR-HPV DNA test applied according to the sampling device and the proportion of adequate samples as determined by amplification of an internal control (a ubiquitous human gene). HPV Selfy assay provides a human beta-globin internal control, used to evaluate sample quality. In the whole study cohort, mean Ct value for the human beta-globin internal control for the HPV Selfy test on self-collected samples, obtained with the direct analysis on self-collected samples, was 26.1 Ct (median value 25.9 Ct, maximum 30.7 Ct, minimum 16.5 Ct). In the subgroup of women with biopsy-diagnosis of cervical lesions CIN2+, the same analysis resulted in 26.7 Ct (median value 26.5 Ct, maximum 30.7 Ct, minimum 24.2 Ct). This means that all women were able to self-collect a similar amount of specimen, confirming self-collected samples’ quality adequacy and so the easiness of the self-sampling procedure using the FLOQSwabs® (Copan, Brescia, Italy).

1. Avian et al. (2022). *Clinical validation of full HR-HPV genotyping HPV Selfy assay according to the international guidelines for HPV test requirements for cervical cancer screening on clinician-collected and self-collected samples.*
2. Meijer et al. (2009). *Guidelines for human papillomavirus DNA test requirements for primary cervical cancer screening in women of 30 years and older.*
3. Arbyn et al. (2018). *VALHUDES: a protocol for validation of human papillomavirus assays and collection devices for HPV testing on self-samples and urine samples.*
4. Tang et al. (2002). *Sample size determination for establishing equivalence/non-inferiority via ratio of two proportions in matched-pair design.*
5. Arbyn et al. (2022). *Can HPV Selfy be considered as a clinically validated HPV test for use in cervical cancer screening?*
