# Supplementary material for: Clinical validation of full HR-HPV genotyping HPV Selfy assay according to the international guidelines for HPV test requirements for cervical cancer screening on clinician-collected and self-collected samples
Source: J Transl Med. 2022 May 17;20:231. doi: 10.1186/s12967-022-03383-x (PMC9115952; doi:10.1186/s12967-022-03383-x)
Supplement: Supplementary file 1 — Additional file 1: Table S1. Intralaboratory reproducibility of HPV Selfy according to Meijer’s guidelines. HPV Selfy assay was performed twice on a subpopulation of 521 samples of which 157 samples positive to HC2. Overall concordance observed was 94.6% (kappa value of 0.87). Table S2. Interlaboratory reproducibility of HPV Selfy according to Meijer’s guidelines. HPV Selfy assay was performed in another laboratory (MediChrom) on a subpopulation of 500 samples of which 150 samples positive to HC2. Overall concordance observed was 93.6% (kappa value of 0.85). Table S3. Intra-laboratory reproducibility of genotype findings of HPV Selfy. Data are presented as number of each genotype detected in each run (i.e. run1 and/or run 2), and numbers do not count up to the total number of HR-HPV positive samples due to multiple infections. [file 12967_2022_3383_MOESM1_ESM.docx]

**Supplementary Information**

**Table S1. Intralaboratory reproducibility of HPV Selfy according to Meijer’s guidelines.** HPV Selfy assay was performed twice on a subpopulation of 521 samples of which 157 samples positive to HC2. Overall concordance observed was 94.6% (kappa value of 0.87).

**Table S2. Interlaboratory reproducibility of HPV Selfy according to Meijer’s guidelines.** HPV Selfy assay was performed in another laboratory (MediChrom) on a subpopulation of 500 samples of which 150 samples positive to HC2. Overall concordance observed was 93.6% (kappa value of **0.85).**

**Table S3.** **Intra-laboratory reproducibility of genotype findings of HPV Selfy.** Data are presented as number of each genotype detected in each run (i.e. run1 and/or run 2), and numbers do not count up to the total number of HR-HPV positive samples due to multiple infections.
